# Inventory of Engineered Nanoparticle-Containing Consumer Products Available in the Singapore Retail Market and Likelihood of Release into the Aquatic Environment

**DOI:** 10.3390/ijerph120808717

**Published:** 2015-07-24

**Authors:** Yuanyuan Zhang, Yu-Rui Leu, Robert J. Aitken, Michael Riediker

**Affiliations:** SAFENANO, IOM Singapore, 30 Raffles Place, #17-00 Chevron House, Singapore 048622, Singapore; E-Mails: Zhang.Yuanyuan@iom-world.sg (Y.Z.); owenleu@hotmail.com (Y.-R.L.); rob.aitken@iom-world.sg (R.J.A.)

**Keywords:** nano-inventory, engineered nanoparticles (ENP), emission quantity, aquatic environment

## Abstract

Consumer products containing engineered nanoparticles (ENP) are already entering the marketplace. This leads, *inter alia*, to questions about the potential for release of ENP into the environment from commercial products. We have inventoried the prevalence of ENP-containing consumer products in the Singapore market by carrying out onsite assessments of products sold in all major chains of retail and cosmetic stores. We have assessed their usage patterns and estimated release factors and emission quantities to obtain a better understanding of the quantities of ENP that are released into which compartments of the aquatic environment in Singapore. Products investigated were assessed for their likelihood to contain ENP based on the declaration of ENP by producers, feature descriptions, and the information on particle size from the literature. Among the 1,432 products investigated, 138 were “confirmed” and 293 were “likely” to contain ENP. Product categories included sunscreens, cosmetics, health and fitness, automotive, food, home and garden, clothing and footwear, and eyeglass/lens coatings. Among the 27 different types of nanomaterials identified, SiO_2_ was predominant, followed by TiO_2_ and ZnO, Carbon Black, Ag, and Au. The amounts of ENP released into the aquatic system, which was estimated on the basis of typical product use, ENP concentration in the product, daily use quantity, release factor, and market share, were in the range of several hundred tons per year. As these quantities are likely to increase, it will be important to further study the fate of ENP that reach the aquatic environment in Singapore.

## 1. Introduction

With the rapid development of nanotechnology, engineered nanoparticles (ENP) are being used in a widening range of industrial sectors and commercial products. ENP are being increasingly used in catalysts, cosmetics and personal care products, pharmaceutical and medical applications, lubricants and fuel additives, paints and coatings, construction materials, chemical and biological sensors, optics and optical devices, food processing and packaging, agrochemicals, textiles and clothing, detergents, security and authentication applications, UV-absorbers and free-radical scavengers, plastics, weapons and explosives, and in countless other products and materials [1,2,3,4]. ENP have also been used for water treatment and environmental remediation (e.g., nano-Fe, TiO_2_, Ag), which may be associated with the release of ENP into aquatic environment [5,6,7]. Several reviews have reported that nanoscale materials are being used in a widening range of industrial sectors and have made estimates of quantities of commercial scale production and use of nanoscale materials. In a review at the early stage of nanomaterial developments, the Royal Society and Royal Academy of Engineering estimated the production of nanomaterials and predicted the quantities used in various application areas such as structural application (e.g., ceramics, catalysts, coatings, powders, and metal) at 10 tons in 2003–2004, and predicted it to increase to 1000 tons by 2010 and between 10,000 and 100,000 tons per year by 2020 [8]. Aitken *et al.*, (2006) indicated that the main applications of nanomaterials in the UK were in catalysts, lubricants and fuel additives, paints, cosmetics and personal care products (e.g., sunscreens), drug delivery, electronics and sensor devices, functional coatings (e.g., on glass, textiles), *etc.* [3]. Schmid and Riediker (2008) reported that Ag, Al-O_x_, Fe-O_x_, SiO_2_, TiO_2_ and ZnO were used in considerable quantities (>1000 kg/year per company) in Swiss industries [9]. A 2010 report by the Dutch government suggested that the number of nano-enabled consumer products had tripled since 2006, though they realized that many of these products were no longer labeled as containing nano [4].

The rapid growth of utilization of ENP in commercial products has triggered concerns on the health and safety, environmental, ethical, policy, and regulatory aspects. These concerns were expressed early on, in the review by the Royal Society and the Royal Academy of Engineering in 2004 [8], and subsequently in many related articles [2,11,12,13]. Consideration of the application patterns suggests that ENP present in a bonded, fixed, or embedded form may not pose a risk to consumers’ health or to the environment during their life cycles. Some applications, on the other hand, may pose a greater potential risk of exposure than others. Of particular concern are those processes, products, and applications that involve large commercial scale manufacturing, use, and disposal of ENP-containing products. Some of these may lead to direct human exposure to ENP via inhalation (e.g., cleaning aids, spray cosmetics, coatings), dermal exposure (e.g., cosmetics), oral ingestion (e.g., food and drink), or intravenous routes (e.g., medicine and diagnostic aids). The estimation of direct exposure in such cases has to some extent been achievable [14,15,16,17]. In contrast, the estimation of indirect exposure (e.g., via drinking ENP-containing water or through the food chain) through environmental routes remains highly challenging. These indirect exposures may arise from a release of ENP into the environment (*i.e.*, air, water and soil) during the manufacturing process, use, and disposal of ENP-containing products (e.g., water treatment facilities, ship exterior paint, food processing, and personal care products).

To date, little is known about the occurrence, fate, and toxicity of ENP in the environment. In part this is due to the lack of sufficiently sensitive and interference-free methodology for the quantitative and qualitative detection of ENP in a complex matrix, such as the water environment [18]. Given these challenges with analytical techniques, it is important to be able to model or estimate the amount of ENP released into different aquatic environments to support the assessment of the risk to human exposure to ENP-contaminated water and to provide information on ENP behavior in water for regulatory decision-making. Estimates of the distribution and usage of ENP-containing consumer products in daily activities, the release routes, and the environmental fate and behaviors of ENP and therefore indirect human exposure to ENP, not only contribute to regulatory decision-making but also allow the development of suitable experimental approaches to test ENP in complex environmental matrices and help define appropriate instrument detection limits. The objective of this project was to understand the distribution and use of ENP in consumer products available in the Singapore retail market through a nano-inventory and to estimate the possible amount of each identified ENP reaching the aquatic environment based on ENP concentrations in consumer products, market share, and estimated release quantities.

## 2. Methods

### 2.1. Nano-Inventory

For the purpose of this study, the definition of ENP was based on the definition of nanomaterials recommended by the European Commission: a natural, incidental, or manufactured material containing particles in an unbound state or as an aggregate or as an agglomerate and where, for 50% or more of the particles in the number size distribution, one or more external dimensions is in the size range 1–100 nm. In specific cases and where warranted by concerns for the environment, health, safety, or competitiveness, the number size distribution threshold of 50% may be replaced by a threshold between 1% and 50% [19]. The inventorization of products containing ENP in the Singapore retail market was carried out through the following steps:

(1) Identify product categories that are likely to contain nanomaterials: The application of ENP in consumer articles described in a selection of recent review articles and reports was first briefly reviewed. The identified use types then guided the subsequent inventorization of products in the Singapore consumer market.

(2) Visit stores to identify products of these categories: This nano-inventory was created by visiting stores of all major retailers in Singapore (NTUC xtra, Giant hyper, Sheng Siong, Cold Storage and Watson), and a series of cosmetic stores. Products purchased by individuals abroad or via the internet were not considered, neither were products used for occupational purposes.

(3) Identify products that are “confirmed” to contain nanomaterials: A product was considered as “confirmed” to contain nanomaterials if either the producer declared the use of nanomaterials on their product’s label; if the ingredients listed suggested that they contain nanomaterials; or if there was a match between the product name with online information on websites of major companies (e.g., Sasa, Sephora, Chanel, L’Oréal, Clarins, Shu Uemura, *etc.*), the statistics database on medical care products of the Singapore Health Science Authority (HSA), or the Woodrow Wilson Institute database [20]. A product was also considered as “confirmed” if composition, proposed properties, and database entry were a match.

(4) Identify products that are “likely” to contain nanomaterials: In cases where the ingredients of a product suggested that it might contain nanomaterials or the proposed properties would make it likely that the product contained nanomaterials, we used expert judgement to assess if the product was likely to contain nanomaterials. This included comparing the information about the product with similar nanomaterials described in the literature and what had been reported about the particle size distribution in such products.

(5) Check remaining products if assignment of the term “unlikely” to contain nanomaterials makes sense. For a product that did not declare any ingredients, we checked if this type of product usually contains nanomaterials. We also checked the promised properties and if they make the product more or less likely to contain nanomaterials. For example, cosmetics containing TiO_2_ that were stated to make the skin look whiter (white skin is a sign of beauty among Asian women) were considered to be “unlikely” because only non-nano TiO_2_ would have this effect.

### 2.2. Determination of Emission of ENP into the Aquatic Environment

ENP can be emitted into the aquatic environment during the use and the disposal of ENP-containing products. In order to determine which kind of ENP is most likely to reach the water systems, we identified release factors from previous studies [21,22,23], by evaluating published information on:

(a) The article’s lifetime;
(b) The ways that ENP is incorporated in the products (*i.e.*, suspended in liquid/solid, surface bound, or in the bulk);
(c) Release pattern (*i.e.*, down the drain, runoff, and incineration and landfill).

The obtained information was then used to define the likelihood of ENP-containing consumer products releasing ENP into the aquatic environment.

The quantity of ENP released into the wastewater collection systems was estimated from the concentration of ENP in products (% by weight), the release quantity of the products (g·capita^−1^·d^−1^), and its market share (%). It was expressed in the form of a mass balance (Equation 1):

(1) $$M_{ENP_{i}}=C_{ENP_{i}}\cdot E_{prod_{i}}\cdot F_{share}$$

where $M_{ENP_{i}}$ = daily amount of ENP i released from a product into the aquatic environment, (g·capita^−1^·d^−1^); $C_{ENP_{i}}$ = concentration of ENP in the product, (% by weight); $E_{prod_{i}}$ = daily per capita quantity of the product reaching the aquatic environment, (g·capita^−1^·d^−1^); $F_{share}$ = market share of ENP-containing product, (%); $C_{ENP_{i}}$ = ENP concentration.

None of the investigated products contained information on ENP concentrations and none of the producers shared such information in a publicly available format. Thus, ENP concentration was estimated from the literature and from the allowable maximum level of ENP in a specific product according to the regulatory standards. If no information could be obtained for a specific product, the ENP concentration was extrapolated based on available information on ENP concentrations in the same or similar products. For example, the allowable maximum level of TiO_2_ in sunscreen is 25% according to EU regulations [24]. Based on this information, sunscreen containing another metal oxide as a UV blocking agent (e.g., ZnO) was assumed to contain up to 25% of this UV-blocking agent.

#### 2.2.1. $E_{prod_{i}}$ Daily Release Quantity

The daily release quantity was calculated from the average product amount used by consumers as described in the literature, and information about the aquatic environments into which they are likely to be released. The proportion of these quantities released into water was described with a “release factor.” This factor was defined as the fraction of ENP released to the drain or urban runoff to the total amount used in each application. It was estimated from usage scenarios and anticipated release patterns. For example, some of the ENP contained in sunscreen may be released when people go swimming. In Singapore, swimming is possible only in pools and in the ocean. Most people have easy access to swimming pools either in their condominium or in nearby public sports centers, whereas for swimming in the ocean they have to travel to the coast. For the present study, 10% of ENP was assumed to be released into the sea while the other 90% was assumed to reach the wastewater stream through swimming pools, the shower, and washing of clothes soiled with sunscreen.

#### 2.2.2. $F_{share}$ Market Share

The market share of ENP-containing consumer products was used for the estimation of the quantity of each ENP reaching different aquatic environments. The market share was estimated from the total number of brands identified in the stores and the number of products in each of the categories in Singapore’s retail market assessed in this survey, and from published information about the global nanotechnology market [25].

Consumer products used for transportation purposes show a very different pattern. Thus, estimates of the quantity of ENP released from tires were determined by using published release factors per vehicle combined with driving distance and traffic statistics published by the Land Transportation Authority of Singapore [26,27].

## 3. Results and Discussion

### 3.1. Types of ENP and Their Application in Consumer Products in the Singapore Retail Market

**Figure 1:**
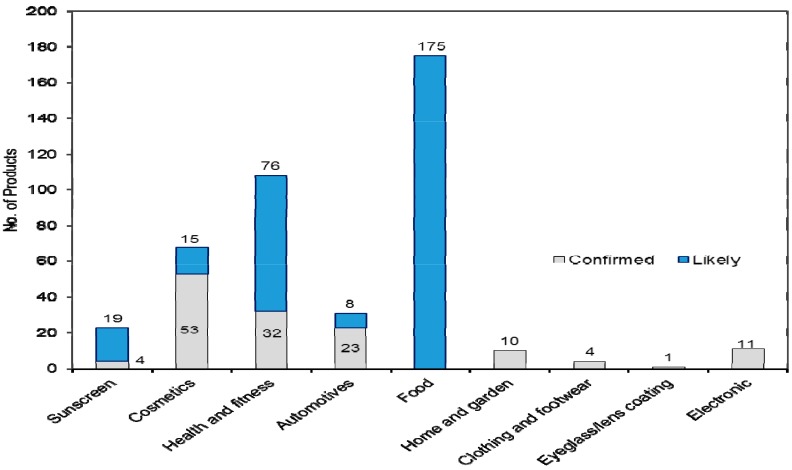
Distribution and category of products with confirmed or likely use of nanomaterials.

In the first step, reports and literature [3,8,9,20,22,28,29,30,31,32,33,34,35,36,37,38,39,40,41], were reviewed to identify the most frequently used ENP and their applications. Ag, TiO_2_, Al/Al_2_O_3_, ZnO, SiO_2_, ceramics, iron/iron oxide, carbon nanomaterials, and nanoclays were identified as the most frequently used ENP in consumer products. The identified ENP along with its function and consumer product application are summarized in Table 1. These identified uses of ENP types guided our nano-inventory activities. In our Singapore market inventory, we evaluated 1432 articles. Of these, 431 were “confirmed” (138 articles) or “likely” (293 articles) to contain ENP. The results are summarized in Table 2. These ENP-containing products occurred in nine different categories: (1) sunscreen, (2) cosmetics, (3) health and fitness, (4) automotive, (5) food, (6) home and garden, (7) clothing and footwear, (8) eyeglass/lens coating, and (9) electronics (see Figure 1). A total of 27 different types of nanomaterials were identified in these articles. In particular, ENP were widely utilized in the category of Food (175 articles), Health and fitness (108 articles), Cosmetics (68 articles), and Sunscreen (23 articles). Of the total of 431 articles, 34 contained more than one type of nanomaterial (e.g., ZnO, TiO_2_, and Al_2_O_3_). In our nano-inventory, it is relevant to note that housekeeping cleaning products supplied in the Singapore market such as laundry detergents or air fresheners have no detailed information regarding the products’ ingredients. Thus, it was not possible to assess if these products contained ENP or not.

**Table 1:** Applications of ENP in identified consumer articles.

ENMs	Function	Application Areas	Reference
Ag	Antimicrobial protection Conductivity or electrical properties	Textiles, food packaging, medical devices, water treatment process, surface coating, electronics such as transparent conducting films, transparent electrodes for flexible devices	[3,8,9,20,28,29,30]
TiO_2_	Absorption of ultraviolet radiation Catalyst	Electronic devices, sunscreen, cleaning, water treatment, solar cell, health and fitness	[8,9,20,28,29]
Al_2_O_3_	Extremely fine powder with great capability of polishing Absorption of light Antimicrobial protection Adsorbent	Cleaning, cosmetics, water treatment, coatings, food additives, catalysts, ceramics, electrical insulators	[8,9,20,28]
Al	Dispersion strength catalyst	Cosmetics, catalysts, coating, optical biomaterials, drug delivery	[8,20,28,31]
ZnO	Sun protection Antimicrobial protection	Sunscreen, cleaning, paints, cosmetics, food packaging, personal care products/sprays	[8,9,20,28]
SiO_2_	Extend life of paints and coatings	Paints, coatings, food packaging	[8,9,20,28]
Zr/ZrO_2_	Good absorbability and hydrophilicity Bio-corrosion resistant and bio-compatible	Drug delivery, water treatment (membrane, ion exchanger), coatings, fuel, battery, pigment, abrasive material, medical implant	[8,28,32,33]
Hydroxyapatite	Biocompatibility	Therapeutic and/or diagnostic agents coatings, drug delivery, sensor, biomaterials	[8,20,28,34,35,36]
Ceramics	Filtration with great rejection efficiency Hardness and strength	Home and health/filter, paint, personal care products, cosmetics, food and beverage, coatings	[3,8,20,28]
CeO_2_	Catalyst Sun protection Optical property	Coatings, paints, automotive/fuel catalysts, biomedicine	[8,28,37,38,39]
Fe/Fe_x_O_y_	Hardness and strength magnetic and catalytic properties	Food and beverage, home and health/sporting goods, environmental remediation, water treatment, catalysts	[8,9,28,40]
Carbon nanomaterials (C_60_, carbon black, carbon nanotubes (CNT)	Hardness and strength Light with high density Conductivity Metallic property Mechanical and Magnetic property	Probing electronic devices, catalysts, adsorbent in water treatment, detection devices, molecular filtration membrane, detection sensor or probe, automotive, sporting goods, clothing, food packaging	[3,8,9,20,22]
Nanoclays	Hardness and strength Improvements in mechanical, thermal, flame resistance, and barrier properties	Food packaging, automotive, cosmetics and toiletries, environment and water treatment, flame retardants, medical devices, packaging, paints, pigments and coatings	[3,8,20,28]
Organic nanomaterials	Organic nanomaterials are widely used in a variety of field, including vitamins, anti-oxidants, color agents, flavors, preservatives, drug delivery, cosmetics, nutrients and supplements.		[8,20,28]
Other nanomaterials	Many other nanomaterials are being increasingly used in the commercial field, including Cu and Cu_x_O_y_, Ti, metal nitrides, and alkaline earth metals. Quantum dots composed of metal (oxide), or semiconductor materials with novel electronic, optical, magnetic and catalytic properties are used in medical imaging, diagnostics and security printing at increasing rates.		[3,8,20,28,41]

In the category of Health and fitness (or personal care products) and Food, TiO_2_ and SiO_2_ were widely used, namely in toothpastes, beverages, instant noodles, and sweets, either as a color additive or an anti-caking agent, respectively. The ingredients of the products had no indication if they were in the nanoscale or not. However, Weir *et al.*, (2012) [42] reported that approximately 39% of food-grade TiO_2_ particles counts were less than 100 nm in at least one dimension and that they readily dispersed into water as fairly stable colloids. Even though this report was limited because the percentage only referred to particles smaller than 220 nm (thus ignoring many of the larger, white TiO_2_ particles), it confirmed the presence of nanoparticles in food-grade TiO_2_. Peter *et al.*, (2012) [43] observed the presence of nanosized SiO_2_ during the digestion of foods containing SiO_2_ as a food additive using SEM. Thus, for the purpose of this study, which aims to estimate ENP concentrations in different aquatic environments, toothpaste, beverages, instant noodles, and sweets were categorized as likely to contain ENP. Ag was used in many consumer products as an antibacterial agent, including: cleaning detergent products in the category of Home and garden (4), household electrical appliances (4) in Electronics, toothbrush (1) in Health and fitness (personal care), and socks (1) in Clothing and footwear.

Of the 431 articles with confirmed or likely use of ENP, producers declared the use of nanomaterials or nanotechnology for 69 of them, but no indication on the type of nanomaterials was provided. They were from the sectors of skincare/makeup/cosmetics (18), health and fitness (23), automotive (22), and clothing (6). Since it was not possible to directly determine the type of nanomaterial, they were not included in the modeling of emission quantity, which may lead to a slight underestimation of quantities.

Most of the commonly used nanomaterials listed in Table 1 were also identified in our inventory of the Singapore retail market. Some others, however, were not found, namely: CeO_2_, Quantum dots, Zr/ZrO_2_, and Hydroxyapatite. This may be attributed to insufficient information about an article’s ingredients, lack of penetration of those types of nanomaterials into the (Singapore) market, or the fact that these may be niche products not found in major retail and cosmetic stores.

### 3.2. Release of ENP during Use and Disposal of Consumer Products

The product types and the ENP contained therein were assessed with regard to their likely release patterns into the aquatic environment during the use or disposal of the articles. The various product categories were assigned to the following release paths:

Sunscreen, cosmetics: Both the review results and nano-inventory results indicate that the main ENP in this category includes TiO_2_ and ZnO, which was expected because sunscreen use was likely to be significant in a tropical area such as Singapore*.* In this category, both TiO_2_ and ZnO, are suspended in liquid. They can be directly released during the use and wash-off process. Most of them will be released into wastewater, while negligible amounts will go into surface water (no swimming in Singapore’s fresh water reservoirs and lakes).

1. Health and fitness (personal care): TiO_2_, SiO_2_, metal oxides, and nano carbon are widely used in this category. Products include: toothpaste, shampoo, shower gel/milk, facial cleaning gel, and body lotion with UV protection. The use of products in this category will lead to direct release of ENP into the wastewater. For example, TiO_2_ can be easily released from toothpaste during tooth brushing. In addition, SiO_2_-containing facial cleaning gel and shampoo have great potential to release particulate SiO_2_ into wastewater.
2. Automotive (*i.e.*, tire, polish and wax, coating, fuel additives): This category was found to be important primarily because of consumables such as tires, which are worn down during their lifetime. This can lead to direct release of ENP into the air and onto road surfaces, from where ENP can go directly into the aquatic environment via road runoff into surface water and in small quantities by air transport and dry deposition. ENP from maintenance products and accessories, such as car coatings or wax products, can be released into road runoff but also into wastewater during their application at private homes. The predominant ENP in this category include carbon black, ceramics, and SiO_2_.
3. Home and garden (e.g., coatings, cleaning products): TiO_2_, Ti, and Ag are widely used in this category. Products in this category are relevant in terms of potential for direct release of ENP into surface water by outdoor use, and into wastewater during indoor use and disposal of products.
4. Clothing and footwear: Ag is used in nano form in textiles as an antimicrobial agent. Wearing clothing with Ag is expected to lead to only minimal direct release of Ag into the aquatic environment. The main release will occur during washing of clothing containing nanoparticles of Ag into the wastewater systems [44]. The amount of ionic and particulate Ag released from the fabrics depends on the type of Ag incorporation into the textile.
5. Eyeglass/lens coating: This is a small-scale use category. ENP may be released into wastewater when washing glasses but only if the ENP were non-permanently coated on the surface of the lenses. The main ENP involved include TiO_2_, SiO_2_, and polymer thin films, which are all permanent coatings.
6. Construction materials, paint, and coating: Most nanomaterials being used in this category are fixed in the products. Some ENP such as Ag will be released in ionic form. Environmental degradation over time and rainfall flushing during heavy storms may cause small quantities of ENP to be released into the aquatic environment. Most of the released nanosized particles are expected to go into surface runoff.

For each of the 431 articles confirmed or likely to contain nanoparticles, the likelihood of releasing ENP into the aquatic environments was assessed based on the above considerations. Of the 431 products assessed, 324 (75%) were identified as likely to release ENP into the aquatic environment and 107 (25%) were unlikely. Table 3 shows the types of matrices that contain ENP in the identified products, the expected release pattern, and the assigned release factor into the aquatic environments. The “release factor” is defined as the fraction of ENP released “down the drain” compared to the total usage quantity. The value of these release factors were identified in the literature and estimated for the purpose of this study. Assumptions behind estimates are listed in the reference column. For example, as women may use facial cottons to remove makeup products such as foundation cream and concealer before face washing, the assumption was made that 70% of the substance was transferred via the facial cottons into solid waste. The remaining 30% will then reach the drain during face washing. In some cases, the value of the release factor was set to 0%, which means these products are not likely to release ENP into the aquatic environment despite the presence of a hypothetical release pathway. For example, nano-capsules and nano-hyaluronic acid in skincare products can be adsorbed when they come into contact with skin. After adsorption, they will lose their nano form and thus the release factor for these skincare products was assumed to be 0%. Also products such as rackets and toothbrushes which have a permanent coating would not release ENP through their lifetime. TiO_2_ and SiO_2_ contained in beverages, snacks, instant noodles, and sweets were assumed to enter into the aquatic environment after passing through the gastrointestinal tract, whereas food that was thrown away would land in solid waste treatment and thus not enter the aquatic system.

**Table 2:** Product category *vs.* ENP within 431 products assessed.

Category	#	TiO_2_	ZnO	Pt	Nano–hyaluronic acid	Si	Cu	Nano capsule	Nano vitamin E	Nano–lipobelle E Q10	Nano Liposome	Au	Ag	SiO_2_	MnO_2_	Ti	Ceramic	Iron oxides	Al_2_O_3_	Alumino–silicate oxides	Copper oxides	Nano Collagen	C_60_	Carbon nanotube	Carbon nanofiber	Nano Fiber	Carbon black	Nano glass	Unclassified
Sunscreen	23	19	12																										
Cosmetics	68	22	2	5	4	1	1	2	1	2	1	12		1	1			4	4	1	1	2				1			19
Health and fitness	108	69	1									1	1	18		2		1					1	1	1	2			22
Automotive	31	1												1			5										1	1	22
Food	175	11	5											159															
Home and garden	10	6											4			1													
Clothing and footwear	4												1																3
Eyeglass/lens coatings	1																												1
Electronics	11												4			4	1												2
Total	431	128	20	6	4	1	1	2	1	2	1	13	10	179	1	7	6	5	4	1	1	2	1	1	1	2	1	1	69

**Table 3:** The type of matrix containing ENP, release pattern and release factor for different products.

Product	Category	ENP Type	Type of Matrix Containing ENP	Release Pattern	Release Factor (%)	Reference
Sunscreen	Sunscreen	TiO_2,_ ZnO	Suspended in liquid	Down the drain	90	Only 0.03% ENP can penetrate into skin after 24 h exposure [45] and 10% loss in the sea
Day cream	Skincare/Cosmetics	Pt	Suspended in liquid	Down the drain	95	Assumption: approximately 5% losses to solid waste and other solid phases.
Facial cleaning gel	Skincare/Cosmetics	Nano-hyaluronic acid	Suspended in liquid	–	0	Based on the assumption: present in form of small vesicles that get approximately 100% adsorbed on the skin and loose nano form.
Facial cleaning gel	Skincare/Cosmetics	MnO_2_	Suspended in liquid	Down the drain	100	Technical guide document on risk assessment, 2003 [46]
Day cream	Skincare/Cosmetics	Au, TiO_2_	Suspended in liquid	Down the drain	95	Assumption: approximately 5% loss to solid waste and other solid phases.
Mask cream	Skincare/Cosmetics	Pt	Suspended in liquid	Down the drain	95	Based on the assumption: approximately 5% loss to solid waste and other solid phases.
Day cream	Skincare/Cosmetics	Nano-capsules	Suspended in liquid	–	0	Nano-capsules were described to be adsorbed completely by Lademann *et al.*, 2013 [47].
Eyeliner	Makeup/Cosmetics	Iron oxides	Suspended in solid	Down the drain	10	90% of the product retention on the facial cotton
Concealer	Makeup/Cosmetics	Al_2_O_3_	Suspended in liquid	Down the drain	30	70% retention on the facial cotton
Foundation powder	Makeup/Cosmetics	ZnO	Suspended in solid	Down the drain	30	70% retention on the facial cotton
Toothpaste	Health and fitness	All	Suspended in liquid	Down the drain	100	Technical guide document on risk assessment, 2003 [46]
Toothbrush	Health and fitness	Ag, Au	Surface bound	Solid waste	0	Permanent coating
Body lotion	Health and fitness	TiO_2_	Suspended in liquid	Down the drain	100	Technical guide document on risk assessment, 2003 [46]
Shower spray	Health and fitness	SiO_2_	Suspended in liquid	Down the drain	80	20% loss during the spray
Shower gel	Health and fitness	ZnO, TiO_2_	Suspended in liquid	Down the drain	100	Technical guide document on risk assessment, 2003 [46]
Shampoo	Shampoo/Health and fitness	TiO_2_	Suspended in liquid	Down the drain	100	Technical guide document on risk assessment, 2003 [46]
Badminton Rackets	Sporting goods	All	Surface bound	Unclassified	0	Disposal of the products as solid wastes
Tire	Automotive	SiO_2,_ Carbon black	Suspended in solid	Runoff	70	Based on the assumption: 70% of the particles deposited on roadsides are washed off of roadsides and reach urban runoff during rainfall.
Car coating (& polish wax)	Automotive	Ceramic, TiO_2_	Suspended in liquid	Down the drain	100	Kojima *et al.*, 2011 [48]
Beverages, instant noodles, seasoning, snacks, sweets	Food	TiO_2_, SiO_2_	Suspended in solid	Down the drain	60	Oral exposure is assumed to be the main route in terms of Food. SiO_2_ and TiO_2_ are assumed to not dissolve during passage through the gastrointestinal tract. However, according to UNEP 2011, 30% of food gets thrown away [49]. Long-term retention in the body is not clear, but assumed to be 10%.
Beverage	Food	ZnO	Suspended in solid	–(as ions)	0	Oral exposure is assumed to be the main route in terms of Food. ZnO is assumed to be transferred into ionic form during passage through the gastrointestinal tract.
Cleaning detergent	Home and garden	TiO_2_, Ag, Ti	Suspended in liquid	Down the drain	100	Technical guide document on risk assessment, 2003 [46]
Filter	Home and garden	Ag	Surface bound	Unclassified	0	Permanent coating
Socks	Clothing and footwear	Ag	Surface bound	Down the drain	100	Technical guide document on risk assessment, 2003 [46]
Hair curling machine	Household appliances/Electronics	Ag, Ti, and ceramic	Surface bound	–	0	Permanent coating
Washing machine	Household appliances/Electronics	Ag	Surface bound	Down the drain	100	Tiede *et al.,* 2011 [50]

### 3.3. Estimation of ENP Reaching the Aquatic Environment

The amount of ENP reaching the aquatic environment was determined from the concentration of ENP in the products containing them, the daily release quantity of this product type to the aquatic environment, and the market share of the products containing ENP.

**Figure 2:**
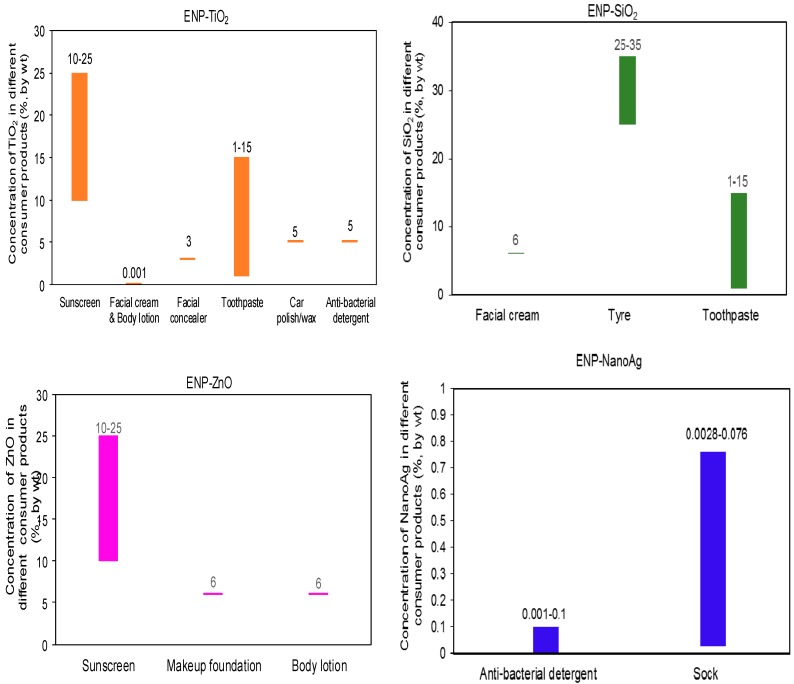
Concentration ranges of nano-scale TiO_2_, SiO_2_, ZnO, and Ag in different consumer product categories (see also Table 4).

*ENP concentrations in products.* The concentration of ENP in different products was estimated from the literature and relevant regulatory standards. Table 4 shows ENP concentrations for different products. Their values were found to vary to some extent among different products of the same category, as shown in Figure 2 for TiO_2_, SiO_2_, ZnO, and Ag. The difference in ENP concentrations is attributed to the different function of ENP in different products.

**Table 4:** Concentrations of nanomaterials in different consumer products.

Category	ENP Type	ENP Concentration (% by Weight)		References
		Low	High	
Sunscreen	TiO_2_	10	25	Weir *et al.*, 2012 [42], EC regulation on Sunscreen [24]
Sunscreen	ZnO	10	25	Weir *et al.*, 2012 [42], EC regulation on Sunscreen [24]
Day cream/Cosmetics	Pt	10	20	Purest colloids: http://www.purestcolloids.com/colloidal-skin-conditioners.php [51]
Day cream/Cosmetics	Nano-lipobelle EQ10	0.5	4	Müller *et al.*, 2007 [52]
Lotion/Cosmetics	Nano hyaluronic acid	60	100	Based on the similar product information: http://www.ebay.com.au/bhp/hyaluronic-acid-serum [53]
Cream/Cosmetics	Nano-capsules	0.5	4	Müller *et al.*, 2007 [52]
Cream/Cosmetics	Au	20	20	Taufikurohmah *et al.*, 2011 [54]
Cream/Cosmetics	SiO_2_	6	15	Tiede *et al.*, 2011 [55]
Facial cleaning gel/Cosmetics	MnO_2_	0.001	0.001	Weir *et al.*, 2012 [42]
Facial cleaning gel/Cosmetics	TiO_2_	0.001	0.001	Weir *et al.*, 2012 [42]
Foundation/Cosmetic	TiO_2_	0.5	4	Müller *et al.*, 2007 [52]
Eyeliner/Cosmetic	Iron oxides	3	3	Tiede *et al.*, 2011 [55]
Eyeliner/Cosmetic	Nano Collagen	0.5	4	Müller *et al.*, 2007 [52]
Concealer/Cosmetic	Al_2_O_3_	3	3	Tiede *et al.*, 2011 [55]
Foundation/Cosmetic	Alumino-silicate oxides	3	3	Tiede *et al.*, 2011 [55]
Eye shadow/Cosmetic	Au	3	3	Tiede *et al.*, 2011 [55]
Foundation/Cosmetic	ZnO	3	3	Tiede *et al.*, 2011 [55]
Toothpaste/Health and fitness	TiO_2_	1	15	Weir *et al.*, 2012 [42], Tiede *et al.*, 2011 [55]
Toothpaste/Health and fitness	SiO_2_	1	15	Weir *et al.*, 2012 [42], Tiede *et al.*, 2011 [55]
Toothbrush/Health and fitness	Ag	0.001	0.1	Weir *et al.*, 2012 [42], Tiede *et al.*, 2011 [55]
Toothbrush/Health and fitness	Au	0.001	0.1	Weir *et al.*, 2012 [42], Tiede *et al.*, 2011 [55]
Body lotion/Health and fitness	TiO_2_	0.001	0.001	Weir *et al.*, 2012 [42]
Shower spray/Health and fitness	SiO_2_	0.001	0.001	Weir *et al.*, 2012 [42]
Shampoo/Health and fitness	TiO_2_	0.001	0.001	Weir *et al.*, 2012 [42]
Tire/Automotive	SiO_2_	25	35	Wik *et al.*, 2005 [56]
Tire/Automotive	Carbon black	25	35	Wik *et al.*, 2005 [56]
Coating/Automotive	Ceramic	10	10	Boxall *et al.*, 2007 [11]
Coating/Automotive	Nano glass	10	10	Boxall *et al.*, 2007 [11]
Coating/Automotive	TiO_2_	5	5	Boxall *et al.*, 2007 [11]
Beverage/Food	SiO_2_	0	<1.5	Health Canada: http://www.hc-sc.gc.ca/fn-an/securit/addit/list/1-anti-eng.php [57]
Beverage/Food	ZnO		<1.5	Health Canada: http://www.hc-sc.gc.ca/fn-an/securit/addit/list/1-anti-eng.php [57]
Beverage/Food	TiO_2_		<1.5	Health Canada: http://www.hc-sc.gc.ca/fn-an/securit/addit/list/1-anti-eng.php [57]
Instant noodle/Food	SiO_2_	1	1	Health Canada: http://www.hc-sc.gc.ca/fn-an/securit/addit/list/1-anti-eng.php [57]
Sweets/Food	TiO_2_	0.34	0.34	European food safety authority, 2004 [58].
Snack/Food	SiO_2_	1	1	Health Canada: http://www.hc-sc.gc.ca/fn-an/securit/addit/list/1-anti-eng.php [57]
Seasoning/Food	SiO_2_	1	1	Health Canada: http://www.hc-sc.gc.ca/fn-an/securit/addit/list/1-anti-eng.php [57]
Cleaning/Home and garden	TiO_2_	5	5	Boxall *et al.*, 2007 [11]
Cleaning/Home and garden	Ag	0.001	0.1	Boxall *et al.*, 2007 [11]
Cleaning/Home and garden	Ti	5	5	Boxall *et al.*, 2007 [11]
Antimicrobial coating/Home and garden	TiO_2_	5	5	Boxall *et al.*, 2007 [11]
Clothing and footwear	Ag	0.0028	0.076	Benn and Westerhoff, 2008 [44]
Household electrical appliances/Electronics	Ag	100	100	Tiede *et al.*, 2011 [55]

In some product categories, several different types of ENP may be used. For example, some manufacturers only added either TiO_2_ or ZnO (up to 25%) to their sunscreen, while others used both. To estimate the individual ENP concentrations, TiO_2_ and ZnO were assumed to contribute equally to the total of 25% w/w contents. This approach was used for all categories, in which an article contained several types of ENP with similar functionality.

*Daily release quantity.* The usage quantity of ENP-containing consumer products was estimated from surveys of personal care products consumption in other countries. Biesterbos *et al.*, (2013) [59] conducted a survey covering every province of the Netherlands. Both females and males were surveyed about usage patterns of personal care products in the past year. A wide range of personal care products were included, such as general hygiene, hair care, skin care, cosmetics (skincare products and makeup), and sunscreen. The collected data were used to determine the daily usage of these personal care products. While the study provided useful average usage data, no information was given about data variation such as standard deviation, range, or percentiles. Hall *et al.*, (2007) [60] conducted a Europe-wide survey that focused on cosmetics but without breaking down the respondents by age. Loretz *et al.*, (2008, 2006, and 2005) [50,61,62] did a survey to investigate the usage of cosmetics and personal care products over two weeks among females in the USA.

The literature review provided useful information but also indicated that the usage quantity for the same product can differ considerably between countries. For example, the per capita (pc) daily usage quantity range for body lotion was 0.4–21.4 (g/pc/d) in the Netherlands [59], 1.84–7.25 (g/pc/d) in Europa [60], and 3.8–13.8 (g/pc/d) in the USA [61]. The difference on the usage quantity among different countries may be attributed to variations in the survey, such as respondents’ age, ethnic group, body shape and gender, individual habits, and weather. To determine the usage quantity for food and beverages in Singapore, we selected data to be as representative as possible for Singapore, as detailed in Table 5. In addition to the abovementioned references, the Technical Guidance Document on Risk Assessment [46], a survey in the Exposure Factors Handbook [63], and the approach used in the UK to estimate nanomaterials’ release into water [55] were also used to collate data on the usage pattern of car coatings/polish, food consumption, cleaning products, and clothing. Table 5 summarizes the usage quantity for each article.

**Table 5:** Summary of daily per capita usage quantity for different products.

Product Name	Category	Usage Quantity (g/pc/d)		
		Low	High	References
Sunscreen	Sunscreen	0.04	1.90	Biesterbos *et al.*, 2013 [59]
Day cream	Skincare/Cosmetics	0.1	1.1	Biesterbos *et al.*, 2013 [59]
Eye cream	Skincare/Cosmetics	0.005	0.055	Assumption: 1/20 of the day cream.
Toner	Skincare/Cosmetics	0.5	4.3	Biesterbos *et al.*, 2013 [59]
Night cream	Skincare/Cosmetics	0.09	0.9	Biesterbos *et al.*, 2013 [59]
Facial cleaning gel	Skincare/Cosmetics	0.5	4.3	Biesterbos *et al.*, 2013 [59]
Cream mask	Skincare/Cosmetics	0.3	3.3	Assumption: 3 times more than day cream
Foundation	Makeup/Cosmetics	0.002	0.132	Loretz *et al.*, 2006 [50]
Foundation powder	Makeup/Cosmetics	0.002	0.132	Loretz *et al.*, 2006 [50]
Eyeliner	Makeup/Cosmetics	0.0002	0.0003	Biesterbos *et al.*, 2013 [59]
Concealer	Makeup/Cosmetics	0.006	0.06	Assumption: 10% of the usage quantity of the foundation.
Eye shadow	Makeup/Cosmetics	0.006	0.06	Assumption: 10% of the usage quantity of the foundation.
Toothpaste	Toothpaste/Health and fitness	1.515	2.669	Hall *et al.*, 2007 [60]
Shampoo	Health and fitness	3.79	21.91	Loretz *et al.*, 2006 [50]
Body lotion	Health and fitness	1.836	7.25	Hall *et al.*, 2007 [60]
Shower gel	Health and fitness	6	23	Loretz *et al.*, 2006 [50]
Tire	Automotive	0.120 (unit: g/km)	0.120	Gehrig *et al.*, 2005 [64]
Coating	Automotive	0.3	0.3	Tiede *et al.*, 2011 [55]
Instant coffee	Beverage/Food	1.0	1.0	Derived from annual instant coffee consumption of 2000 tons in Singapore [65]
Instant noodles	Food	3.6	3.6	Seasoning powder (15 g) is 14% of an instant noodle package weight (110 g). Daily intake of grain products is 3.7 g/kg-day, cited from 2011 Exposure Factors Handbook of the USEPA [63]. The range of BMI for Asians is 17–35 kg/m^2^ (WHO expert consultation report, 2004) [66]. The average height for male and female in Singapore is 1.7 and 1.6 meters, respectively [67]. Therefore, the estimated average body weight for Asians is assumed to be 70 kg. The percentage of instant noodles in total grain products is assumed to be 10%. Therefore, daily intake of instant noodles is 14% × 259 × 10% = 3.6 g.
Seasoning powder	Food	0.032	0.032	Carlsen *et al.*, 2011 [68]
Snacks (Chips)	Food	0.42	0.98	Daily intake of snacks for Asians is 0.1 (± 0.04) g/kg-day (USEPA, 2011, Exposure factors handbook) [63]. The estimated average body weight for Asians is assumed to be 70 kg. The percentage of chips in total snacks is assumed to be 10%. Daily intake of chips is = 7(± 2.8) × 10% = 0.7(± 0.28) g.
Sweets (Chocolate candy)	Food	0.14	0.14	Daily per capita intake of sweets is 0.4 g/kg-day in Exposure Factors Handbook [63]. The estimated average body weight for Asians is assumed to be 70 kg. The percentage of chocolate candy investigated in our inventory in total sweets is assumed to be 5%. Individual daily intake of chocolate candy is 0.4 × 70 × 5% = 0.14 g.
Cleaning	Home and garden	110	110	Technical guide document on risk assessment, 2003 [46]
Clothing	Clothing and footwear	4.45	4.45	Assumption: 1/20 of emission from clothing. Tiede *et al.*, 2011 [55]
Washing machine	Household electrical appliances	1.375	1.375	Tiede *et al.*, 2011 [55]

*Market share.* The market share for each product category was estimated from our market survey and from published data, as shown in Table 6.

**Table 6:** Estimated market share of ENP-containing consumer products in the Singapore retail market (“Market survey” refers to our assessments conducted in local stores).

Category	Market Share (%)	Reference
Sunscreen	85	Market survey: out of 27 products, 23 are likely to contain ENP.
Skincare/Cosmetics	1	Tiede *et al.*, 2011 [55]
Makeup/Cosmetics	2	Tiede *et al.*, 2011 [55]
Toothpaste/Health and fitness	78	Market survey: Out of 79 toothpaste, 62 are likely to use ENP.
Hair treatment/Health and fitness	3	Market survey: Out of 309 products 9 likely to contain ENP
Body lotion/Health and fitness	0.5	Tiede *et al.*, 2011 [55]
Shower spray/Health and fitness	0.1	Tiede *et al.*, 2011 [55]
Shower gel/Health and fitness	2.5	Market survey: Out of 162 products, 4 are likely to contain ENP.
Tire/Automotive	10	Tiede *et al.*, 2011 [55]
Coating/Automotive	<1	Tiede *et al.*, 2011 [55]
Beverages/Food	31	Market survey: Out of 148 products, 46 are likely to use ENP.
Instant noodles/Food	26	Market survey: Out of 232 products, 60 are likely to use ENP.
Seasoning/Food	0.1	Based on the assumption: market share is very low, 0.1%.
Snack/Food	21	Market survey: Out of 247 products, 53 are likely to use ENP.
Sweets/Food	13	Market survey: Out of 79 products, 10 are likely to use ENP.
Cleaning/Home and garden	<1	Tiede *et al.*, 2011 [55]
Clothing and footwear	<1	Tiede *et al.*, 2011 [55]
Household electrical appliances	<1	Tiede *et al.*, 2011 [55]

*Emission quantity of ENP.* Data on ENP concentrations, release quantity, and market share were combined to estimate the emission quantity of each ENP using Equation 1. Table 7 shows the emission quantities of ENP reaching different environmental compartments (*i.e.*, water, air, and soil). For the aquatic environment, municipal sewerage and urban runoff are the two main ENP-accepting bodies in Singapore. The most important ENP emitted into municipal sewage were, from high to low quantity: TiO_2_, ZnO, SiO_2_, Ag, and Au. For urban runoff, carbon black and SiO_2_ were the major components that could reach the urban runoff collection systems (e.g., collection ponds, channels, and eventually reservoirs).

In addition to entering into the aquatic environment, ENP can also be transported to soil and air compartments during the use and disposal of the products (see Table 7 for the respective release factors). Solid waste is incinerated in Singapore and only a very small portion of ENP can pass through these modern incineration plants with state-of-the-art flue gas washing and effective filtration systems [69]. The ash residue in which any remaining ENP may be collected is then transported to a landfill on Semakau Island, thus they are removed from Singapore’s mainland. The aquatic environment is the main accepting body for the released ENP identified in this study.

The daily per capita release quantities obtained above can be multiplied by the number of people living in Singapore (5.3 million in 2013 [70]) or, in the case of automotive products, the number of registered vehicles (969,910 in 2012 [27]) to obtain the total quantity of ENP released annually into the aquatic environment of Singapore. The estimated ranges of release of the predominant ENP are: TiO_2_ (73.5 to 969.0 t/y), followed by ZnO (1.2 to 272.0 t/y), SiO_2_ (40.9 to 196.0 t/y thereof 21.2 to 29.7 t/y from road surface runoff), carbon black (21.2 to 29.7 t/y, mostly from runoff), Ag (26.7 to 27.5 t/y), and Au (0.002 to 2.600 t/y).

**Table 7:** Summary of quantity of ENP reaching different environmental compartments.

No.	ENP Type	Product/Category	Release into Water	Emission Quantity (g/pc/d)		Release Elsewhere	Emission Quantity (g/pc/d)	
				Low	High		Low	High
1	TiO_2_	Sunscreen, automotive, cleaning, skincare, makeup, toothpaste, shampoo, body lotion, shower gel, beverages	Down the drain	3.80E–02	5.01E–01	Losses in the sea, solid waste	2.30E–04	2.50E–02
2	ZnO	Sunscreen, makeup, skincare	Down the drain	5.93E–04	1.41E–01	Solid waste	6.59E–05	1.56E–02
3	Pt	Skincare	Down the drain	7.20E–07	3.17E–04	Solid waste	3.79E–08	1.67E–05
4	Au	Skincare, makeup	Down the drain	1.21E–06	1.36E–03	Solid waste	2.25E–07	7.33E–05
5	MnO_2_	Skincare	Down the drain	1.52E–09	1.30E–08	N/A	N/A	N/A
6	SiO_2_	Skincare, toothpaste, shower spray, beverages, instant noodles, seasoning, snacks	Down the drain	1.02E–02	8.59E–02	Solid waste and aerosol in the air	4.12E–03	9.14E–03
7	Alumino–silicate oxides	Makeup	Down the drain	1.80E–08	1.19E–06	Solid waste	4.20E–08	2.77E–06
8	Iron oxides	Makeup	Down the drain	3.00E–08	9.00E–06	Solid waste	2.70E–07	8.10E–05
9	Al_2_O_3_	Makeup	Down the drain	2.40E–08	7.20E–06	Solid waste	5.60E–08	1.68E–05
10	Cu	Makeup	Down the drain	1.08E–08	1.08E–07	Solid waste	2.52E–08	2.52E–07
11	Si	Makeup	Down the drain	1.08E–08	1.08E–07	Solid waste	2.52E–08	2.52E–07
12	Collagen	Makeup	Down the drain	2.00E–10	2.40E–09	solid waste	1.80E–09	2.16E–08
13	Carbon black *	Tire	Runoff	5.98E–02	8.38E–02	Other environmental compartments: air and soil.	2.56E–02	3.59E–02
14	SiO_2_ *	Tire	Runoff	5.98E–02	8.38E–02	Other environmental compartments: air (airborne particles) and soil.	2.56E–02	3.59E–02
15	Ceramic	Car coating	Down the drain	1.20E–04	1.20E–04	N/A	N/A	N/A
16	Nano glass	Car coating	Down the drain	3.00E–05	3.00E–05	N/A	N/A	N/A
17	Ti	Home cleaning	Down the drain	5.50E–03	5.50E–03	N/A	N/A	N/A
18	Ag	Cleaning, clothing and footwear, household electrical appliances	Down the drain	1.38E–02	1.42E–02	N/A	N/A	N/A

* Unit: g/car/d.

## 4. Conclusions

The nano-inventory of consumer products supplied in the Singapore retail market confirms that nanoparticle-enabled products have reached the market in sizeable quantities. The investigations were done in major Singaporean retail stores and completed through Internet searches on frequently used commercial platforms. Thus, this survey provides a good estimate of the market distribution and the use of ENP-containing consumer products in Singapore. The assessment of the potential of the various ENP being released into different aquatic environments throughout their lifetime suggests that the majority is released into wastewater and urban runoff. TiO_2_ and SiO_2_ are the most important ENP released into wastewater, whereas “traditional” ENP from tire wear is the main contributor to ENP reaching the urban runoff in Singapore.

Singapore treats all waste water and it recently introduced a program to let urban runoff pass through elementary treatment cells (e.g., vegetated swale or ponds, wetlands) for purification of this runoff and to reduce peak flow rates of related pollutants [71,72]. Thus, both release routes will face treatment processes. The emission quantities determined in this study represent the initial amount of ENP reaching the aquatic environment before these treatment processes in wastewater/water plants, where most of ENP will be removed from water [73,74,75,76]. The information presented here about ENP and their target water bodies represents a useful starting point and can be used for modeling the fate of ENP in the aquatic environment of Singapore.
